# Magnitude and Factors Associated with Gestational Weight Gain Adequacy among Pregnant Women in South Gondar Zone, Northwest Ethiopia

**DOI:** 10.1016/j.cdnut.2023.102031

**Published:** 2023-11-14

**Authors:** Melaku Tadege Engidaw, Alemayehu Digssie Gebremariam, Bedilu Abebe Abate, Desalegn Tesfa, Sofonyas Abebaw Tiruneh, Yenehun Addisu, Yismaw Yimam Belachew

**Affiliations:** 1 Public Health Department, College of Health Sciences, Debre Tabor University, Debre Tabor, Ethiopia; 2 Public Health, School of Medicine and Dentistry, Gold Coast Campus, Griffith University, Gold Coast, Queensland, Australia; 3 Department of Gynecology and Obstetrics, School of Medicine, College of Health Sciences, Debre Tabor University, Debre Tabor, Ethiopia

**Keywords:** Magnitude, weight gain adequacy, prepregnancy BMI, pregnant women, Ethiopia

## Abstract

**Background:**

Weight gain during pregnancy depends on the maternal prepregnancy weight and height. Inappropriate weight gain has negative consequences, including the health care system and society because of its adverse birth outcomes.

**Objective:**

This study aimed to assess the magnitude and factors associated with gestational weight gain in Northwest Ethiopia.

**Methods:**

From September 2018 to June 2019, a community-based prospective follow-up study was conducted in Northwest Ethiopia. A total of 422 pregnant women were followed from conception to delivery and the data were collected using a multistage sampling technique. Stata 14 standard edition (SE) software was used for data analysis. Multinomial logistic regression was used to determine the relationship between dependent and independent variables. *P* value of ≤0.05 was used to determine statistical significance.

**Results:**

Majority of the participants had normal weight gain [65.12%, 95% confidence interval (CI): 60.08, 69.85]. Besides this, the rate of inadequate and overadequate weight gain was 21.53% (95% CI: 17.60, 26.05) and 13.35% (95% CI: 10.22, 17.25), respectively. Inadequate weight gain was linked to meal frequency [adjusted odd ratio (AOR): 0.52, 95% CI: 0.28, 0.97], targeted supplementary feeding program (TSFP) enrollment (AOR: 2.47; 95% CI: 1.35, 4.50), parity (AOR: 0.18; 95% CI: 0.05, 0.62), and alcohol consumption history (AOR: 0.47; 95% CI: 0.25, 0.88), whereas overadequate weight gain was associated with residency (AOR: 5.22; 95% CI: 2.43, 11.22) and TSFP status (AOR: 2.22; 95% CI: 1.08, 4.57).

**Conclusions:**

This study revealed a notable magnitude of both inadequate and overadequate weight gain. It found that good meal frequency, alcohol consumption, parity, and TSFP enrollment were associated with a reduced risk of inadequate weight gain during pregnancy. In addition, the study identified residence and TSFP enrollment as factors linked to overadequate weight gain during pregnancy.

## Introduction

Weight gain during pregnancy is the increment of maternal weight by 20% of her prepregnancy weight because of the formation and building up of tissues and body fluids [1]. According to the Institute of Medicine (IOM) recommendation, weight gain adequacy is the amount of net total weight gain at the end of 9 month of pregnancy based on the prepregnancy body mass index (BMI). If a woman with a prepregnancy BMI of 18.5 kg/m^2^, 18.5–23.9, 24.0–27.9, and >28 gains 12.5–18 kg, 11.5–16 kg, 7–11.5 kg, and 5–9 kg correspondingly, her weight increases during pregnancy [2]. Similar to others, 27% of the productive age women in Ethiopia had undernutrition/underweight (BMI <18.5). Insufficient nutrient intake, indicated by a low BMI during pregnancy, childhood, and adolescence, has been associated with adverse birth outcomes and obstetric complications [3].

According to IOM, the common factors associated with weight gain adequacy are sociodemographic, behavioral/lifestyles, psychosocial, medical conditions, and the existence of medical care, which is very important to reexamine in a developing country context [2,4].

Generally, inadequate weight gain predisposes the fetus to have small-for-gestational age (SGA), low-birth weight and preterm delivery [5]. An overadequate weight gain leads to maternal health problems, such as infertility, postpartum complications, gestational diabetes, pre-eclampsia, cesarean section, and fetal macrosomia/large-for-gestational age (LGA) [6,7]. Even if the overall magnitude of inappropriate weight gain worldwide is not estimated, it is very high in some countries, such as Indonesia and Canada. Also, there is limited study in sub-Saharan African countries [8,9].

Weight gain is the most significant factor for healthy fetal growth in each day, week, and trimester [10]. Women in the reproductive age group enter into pregnancy with a poor nutritional status, which leads to insufficient weight gain during pregnancy that will contribute to confrontational adverse health outcomes for both the mothers and the fetus [8]. Appropriate weight gain during pregnancy will depend primarily on the prepregnancy BMI. Age, educational status, ethnicity, smoking, hypertension, diabetic mellitus [11,12], age at first marriage, dietary habits, food security conditions, and antenatal care (ANC) follow-up [13].

The common complication because of inappropriate weight gains was associated with preterm delivery, too large infant at birth, preterm birth, and childhood obesity and conditions that are prone to chronic diseases in the later life of the fetus [[14], [15], [16]]. An over or underweight woman will face preterm delivery, SGA, and low-birth weight infants. Whereas, in this study, the incidence of gestational diabetes mellitus, pregnancy-induced hypertension, and LGA/macrocosmic infants were rare [[17], [18], [19], [20]].

According to a population-based study in the United States, 32.0% gained weight within guidelines, whereas 20.9% gained inadequately and 47.2% gained excessive weight. Also, the highest prevalence of inadequate weight gain (39.3%) was found among underweight women, whereas overweight and obese class I women had the highest magnitude of excessive weight gain (64.1% and 63.5%, respectively) [12]. In another study, weight gain was lower than the recommendation of IOM (17.6%). In addition, the rate of inadequate weight gain was 17.2%, 22.0%, 6.1%, and 14.3% among underweight, normal weight, overweight, and women with obesity, correspondingly [9].

A systematic review in sub-Saharan African countries revealed that the adequacy of gestational weight gain (GWG) varied significantly, ranging from 3% to 62%. Also, more inadequate weight gain was >50%. For underweight women, the range of inadequate weight gain was even higher, extending from 67% to 98% [21].

In Indonesia, 16.7% of the women had chronic energy deficiency before pregnancy. The mean ± standard deviation (SD) of the total weight gain for all pregnant women was 8.3 ± 3.6 kg. Of all, 79% did not meet the recommended weight gain related to their prepregnancy BMI. The rate of weight gain was highest during the second trimester as compared with other trimesters. And the total weight gain was associated with prepregnant BMI, education, and socioeconomic status [8].

In India, 59% of pregnant women in the second trimester had a weight gain of <60 g/d after 2 wk of measurement, and 53% of pregnant women in the third trimester had a weight gain of <54 g/d as per recommendations. There was no association between weight gain patterns during the second and the third trimester of pregnancy and availing antenata care (ANC) [10]. Because most of the studies were conducted in wealthy countries, identifying possible factors that influence recommended weight increase during pregnancy and measuring the quantity in the context of low-socioeconomic levels, such as Ethiopia, is critical. Nevertheless, women with poor nutritional status are a severe problem in developing nations [3].

So, this study aimed to assess maternal weight gain adequacy and its associated factors in the study area, which is vital to develop strategies to mitigate the problem and enhance the well-being of the mother and the fetus. It is also essential to tackle the intergenerational effect of malnutrition in the life cycle through appropriate nutritional assessment and counseling before and during pregnancy.

## Methods

### Study area

This study was conducted in the South Gondar Zone. South Gondar Zone is located 666 km to the North of Addis Ababa, the capital of Ethiopia. According to the 2007 census, the zone has a population of 2,047,206 (1,038,913 were males and 1,008,293 were females) with a population density of 145.56. This zone has 10 Woreda and 2 Town administrations with a total of 378 Kebeles at the time of data collection. At the time of data collection, there are 93 Health Centers, 3 primary hospitals, and 1 comprehensive hospital to serve the community with other health extension packages in health posts.

### Study design and period

Community-based prospective longitudinal study was conducted from September 2018 to June 2019 in the South Gondar Zone, Northwest Ethiopia.

### Study population

The study population was all reproductive age group women who were married or had a partner and had menstruation within 1 month before the census time. Furthermore, participants were selected if they had a plan to be pregnant soon and were permanently residing in the study area. In contrast, the pregnant and unmarried/without a life partner (hence, culturally unsuitable for pregnancy monitoring) women were excluded from this study. Also, if a woman had a recent history of different contraceptive methods utilization, and at the age of menopause were not included in this study. After the census, all women who became pregnant within 6 month after the survey were included in this study.

### Sample size determination

The sample size was calculated using the single population proportion formula using a 5% margin of error and a 95% confidence level and the proportions of adequate, inadequate, and overadequate weight gain were 32.0%, 20.9%, and 47.2%, respectively [9].

The sample sizes for acceptable (*n*_*a*_), inadequate (*n*_*i*_), and overadequate (*n*_*o*_) weight gain were 334, 254, and 383, respectively. After a 10% no-response rate and taking the largest sample size, the final sample for this study was 422 pregnant women.

### Sampling procedure

In the South Gondar Zone, there are 12 Woreda/districts; 3 of them were selected using simple random sampling (Farta district, Layi Gayint district, and Sede – Muja district). Finally, participants were included using a simple random sampling technique after the assessment of eligibility through the house-to-house census. The numbers of women sampled from the selected Kebeles were determined using proportional allocation to the population size of each Kebeles. Of all eligible women, 422 women who were married or had a life partner were registered during census time. After this, weight was measured from prepregnancy till 9 mo of pregnancy. Prepregnancy weight and height measurements were taken cross-sectionally from September to December 2018. After this, a follow-up system was established. In the case of >1 eligible respondent within 1 house, only 1 respondent was selected using the lottery method.

### Variables of the study

#### Dependent variables.

1) Achievement of recommended weight gain during pregnancy (adequate, inadequate, and overadequate).

#### Independent variables.

1) Sociodemographic variables: age, educational status, marital status, religion, residency, ethnicity, monthly income, and occupation.
2) Obstetrics and gynecology-related variables: parity, gravidity, ANC follow-up, postnatal care visits, place, and mode of delivery.
3) Maternal medical history: diabetes mellitus (DM), hypertension, HIV/AIDS, and asthma.
4) Nutrition-related factors: dietary diversity score, midupper arm circumference (MUAC), and prepregnancy BMI.

### Definition of terms

Total weight gain was calculated as the difference between prepregnancy weight and weight at the end of 9 mo [9].

Weight gain adequacy was the net total weight gain as compared with the IOM recommendation, which is categorized as adequate, inadequate, and overadequate if it was within, below, and above the IOM recommendation [9].

BMI was calculated by dividing the weight (kg) of the women to height in meter square (chronic energey defficiency (CED); <18.5, normal; 18.5–24.9, and overweight; ≥25, and obese; ≥30) [22].

### Data collection and quality control

Twelve trained data collectors performed a house-to-house census in selected Kebeles in the South Gondar Zone (Health Extension Workers by profession). The data were collected 10 times starting from the census (from September 2018) to the next 2 year monthly. After identification, interviewer-administered structured questionnaires were used by data collectors.

The trained data collector visited the selected women every month throughout the pregnancy period to conduct the interview and took the anthropometric measurements. Body weight was measured with a calibrated electronic Seca scale that was accurate to 0.1 kg while subjects were wearing the possible lightest cloth. And the height was measured once with a Stadiometer, accurate to 0.1 cm. MUAC was measured on the left arm for right-handed or vice versa by inserting arm circumference tape accurate to 0.1 cm. Unlike height and MUAC, the weight was measured from prepregnancy to 9 month of gestation.

Training on the questionnaire, anthropometric measurement, and standardization were done for the data collectors. The questionnaire was first prepared in English and translated back to the Amharic language. The Amharic version was retranslated back to English again to maintain its consistency. The questionnaire was pretested on 5% of the sample size in similar settings outside the study area before the actual data collection to ensure the clarity of the questionnaire. After the pretest, the necessary changes were employed.

### Data processing and analysis

All returned questionnaires were checked for completeness and consistency of responses and then the data were cleaned, verified, codded, and categorized. After this, the data were entered into Epi-Info 7.0 and analyzed using Stata version 14 (SE) for Windows. In this context, the wealth index was derived using the principal component analysis, and subsequently, we ranked it into 5 categories, ranging from the poorest to the richest. The data were explored for both descriptive and analytic analysis. The association between dependent and independent variables was determined using multivariate (multinomial) logistic regression. A variable having a *P* value of ≤0.05 was considered a statistically significant predictor.

## Ethical approval and consent to participate

Ethical clearance was approved by the Ethical Review Board members of Debre Tabor University (Ref number: DTU/RPD/10/2017). Then, a permission letter was obtained from the respective district, Kebeles administration office and health institutions of respective health office units. Written informed consent was obtained from each study participant after a clear explanation of the purpose of the study. During the data collection period, counseling was employed for women with abnormal BMI results, dietary habits, and the importance of appropriate weight gain. The interview was conducted in a quiet separate room at the first contact. Confidentiality of the information was assured by removing personal identifiers during data entry and management. And the privacy of the respondents was maintained during the interview and physical measurements.

## Result

### Sociodemographic characteristics of the study

In this study, from 422 participants, 367 pregnant women data were analyzed. The nonresponse rate was 13.03%. The mean (±SD) age of the participants was 29.75 (±5.40) yr. More than three-fourth of the respondents were from rural parts. Two-thirds of the pregnant women were not able to read and write. The mean ± SD of the family size of the household was 4.37 ± 1.68 (Table 1).

**TABLE 1:** Sociodemographic characteristics of pregnant women in South Gondar Zone, Northwest Ethiopia, 2019 (*n* = 367)

Characteristics		Frequency	Percentage
Residency	Preurban	83	22.62
	Rural	284	77.38
Current age	18–24	48	13.08
	25–29	127	34.60
	30–34	100	27.25
	35–43	92	25.07
Religion	Orthodox	285	77.66
	Muslim	82	22.34
Family size (mean)	≤4	194	52.86
	>4	173	47.14
Maternal educational status	Unable to read and write	244	66.49
	Able to read and write	68	18.53
	Primary education	17	4.63
	Secondary education	25	6.81
	College and above	13	3.54
Husband’s educational status (364)	Unable to read and write	182	50.00
	Able to read and write	116	31.59
	Primary education	16	4.40
	Secondary education	28	7.69
	College and above	25	6.32
Maternal occupation	Housewife	257	70.03
	Farmer	72	19.62
	Government employer	11	3.00
	Others^1^	27	7.36
Husband’s occupation	Farmer	269	73.90
	Daily laborer	14	3.85
	Government employer	37	9.62
	Merchants	18	4.67
	Others^1^	29	7.97
Wealth index	Very poor	73	19.89
	Poor	75	20.44
	Medium	73	19.89
	Rich	75	20.44
	Richest	71	19.35

1 Others = carpenters, guards, construction workers, nongovernmental workers, and students.

### Reproductive health and medical history-related characteristics

The mean age ± SD at first pregnancy was 19.65 ± 2.78 yr, and the minimum age was 15 yr. More than half of the pregnant women got pregnant for the first time between 15 and 19 yr. The number of average pregnancies was 2.35 (±1.71). In this context, all study participants were free from known chronic diseases, such as asthma, DM, hypertension etc. Most respondents were multi-Para [211 (57.49%)], and 358 (97.55%) of them had ANC visits for the current pregnancy (Table 2).

**TABLE 2:** Obstetrics-related characteristics of the pregnant women in South Gondar Zone, Northwest Ethiopia, 2019 (*n* = 367)

Variable	Categories	Frequency	Percent
Age at first pregnancy	15–19	195	53.13
	20–24	137	37.33
	25–29	35	9.54
Gravidity	Primigravida (= 1)	58	15.80
	Multigravida (2–4)	211	57.49
	Grand gravida (>4)	98	26.70
Parity	≤4	321	87.47
	>4	46	12.53
ANC visits for current pregnancy	Yes	358	97.55
	No	9	2.45

Abbreviation: ANC, antenatal care.

### Health and nutritional status-related factors

None of the respondents had diseases known before (chronic and acute) or dietary restrictions during this study. Because of their pregnancy, 157 (42.78%) pregnant women were a part of targeted supplementary feeding. The mean ± SD of weight and height of respondents before pregnancy was 49.31 ± 5.14 kg and 153.44 ± 5.72 cm, respectively. Also, the mean ± SD of BMI was 20.96 ± 2.02 kg/m^2^. The magnitude of being underweight before pregnancy using BMI was 54 (14.71%). The level of undernutrition during pregnancy by using MUAC (≤22 cm) was 150 [40.87%, 95% confidence interval (CI): 35.93, 46.00].

### Level of weight gain adequacy

Weight gain adequacy was assessed using the first months of pregnancy weight and the height measured during the census. The average weight gain ± SD was 13.85 ± 3.25 kg. From all, 239 (65.12%; 95% CI: 60.08, 69.85) of the pregnant women had adequate (normal) weight gain. As compared with the IOM recommendation, the prevalence of inadequate (underweight) gain was 21.53% (95% CI: 17.60, 26.05), whereas overadequate weight gain was 13.35% (95% CI: 10.22, 17.25). Here, 22 study participants' data were incomplete (abortion and transferring into other areas).

**FIGURE 1:**
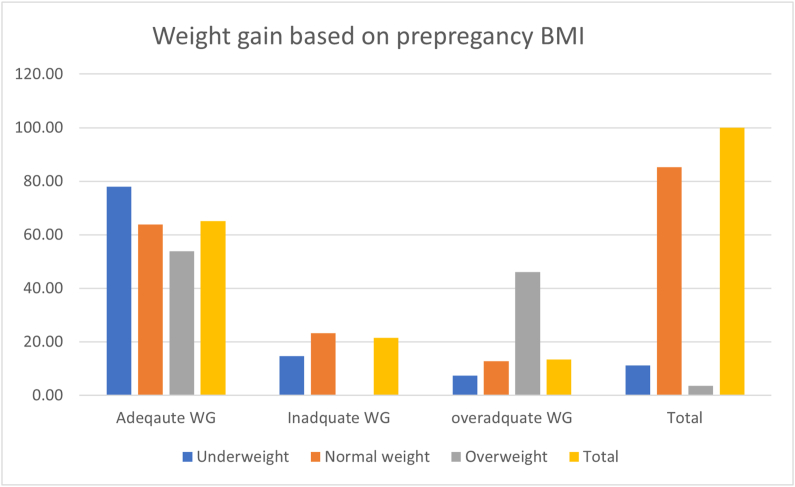
Maternal weight gain magnitude during pregnancy based on pre-pregnancy BMI in South Gondar Zone, Northwest Ethiopia, 2019 (n = 367).

As depicted in Figure 1, of 42 underweight women, 32 of them gained the recommended weight during the pregnancy period. Similarly, among 313 women with normal BMIs, 200 of them achieved the recommended weight gain. In addition, among 13 overweight women during prepregnancy, 7 of them attained the recommended weight gain.

### Factors associated with weight gain adequacy

A bivariate multinomial logistic regression was employed to determine the independent effects of variables. At this time, residence, meal frequency, enrollment in targeted supplementary feeding program (TSFP), prepregnancy BMI, enrollment to Productive SafetyNet Program, the number of live births (Parity), and alcohol intake history during pregnancy were significantly associated with inadequate weight gain. When these variables were combined in a multivariable multinomial logistic regression model, inadequate weight gain during pregnancy was associated with meal frequency, TSFP enrollment, parity, and alcohol consumption history during pregnancy. On the other hand, residence area and enrollment of the TSFP were the contributing factors to the development of overadequate weight gain during pregnancy (Table 3).

**TABLE 3:** Multivariate multinomial logistic regression analysis results of pregnant women in South Gondar Zone, Northwest Ethiopia, 2019 (*n* = 367)

Variables		WGA, *n* (%)			Inadequate vs. adequate		Overadequate vs. adequate	
		Inadequate	Adequate	Overadequate	COR (95% CI)	AOR (95% CI)	COR (95% CI)	AOR (95% CI)
Residence	Rural	199 (83.26)	61 (77.22)	24 (48.98)	1	1	1	1
	Urban	40 (16.74)	18 (22.78)	25 (51.02)	1.47 (0.78, 2.74)	1.39 (0.67, 2.85)	5.18 (2.69, 9.97)	5.22 (2.43, 11.22)^1^
Meal frequency	Low	125 (52.30)	47 (59.49)	20 (40.82)	1	1	1	1
	Good	114 (47.70)	32 (40.51)	29 (59.18)	0.74 (0.44, 1.25)	0.52 (0.28, 0.97)^1^	1.59 (0.85, 2.96)	0.79 (0.37, 1.65)
Enroll in TSFP	No	158 (66.11)	34 (43.04)	18 (36.73)	1	1	1	1
	Yes	81 (33.89)	45 (56.96)	31 (63.27)	2.58 (1.53, 4.34)	2.47 (1.35, 4.50)^1^	3.36 (1.77, 6.36)	2.22 (1.08, 4.57)^1^
Prepregnancy BMI	Underweight	39 (16.32)	6 (7.59)	9 (18.37)	1	1	1	1
	Normal	200 (83.68)	73 (92.41)	40 (81.63)	2.37 (0.96, 5.83)	2.40 (0.93, 6.17)	0.87 (0.38, 1.92)	0.94 (0.38, 2.31)
Part of PSNP	Yes	42 (17.57)	20 (25.32)	13 (26.53)	1.59 (0.87, 2.91)	1.08 (0.53, 2.20)	1.69 (0.82, 3.46)	1.86 (0.80, 4.35)
	No	197 (82.43)	59 (74.68)	36 (73.47)	1	1	1	1
Parity	≤4	202 (84.52)	76 (96.20)	43 (87.76)	0.21 (0.06, 0.71)	0.18 (0.05, 0.62)^1^	0.76 (0.30, 1.91)	1.08 (0.38, 3.04)
	>4	37 (15.48)	3 (3.80)	6 (12.24)	1	1	1	1
History of taking alcohol	No	99 (41.42)	18 (22.78)	14 (28.57)	1	1	1	1
	Yes	140 (58.58)	61 (77.22)	35 (71.43)	0.42 (0.23, 0.74)	0.47 (0.25, 0.88)^1^	0.56 (0.29, 1.10)	0.73 (0.35, 1.53)

Abbreviations: AOR, adjusted odd ratio; CI; confidence interval; COR, crude odd ratio; PSNP, Productive SafetyNet Program; TSFP, Targeted Supplementary Feeding Program; WGA, weight gain adequacy.

1 = Reference.

^1^Significant at *P* ≤ 0.05.

### Inadequate weight gain related to adequate weight gain adequacy

If a participant increased their weight gain rate by one unit, the odds of having inadequate weight gain related to adequate weight gain would be reduced by a factor of 0.52 [adjusted odd ratio (AOR): 0.52; 95% CI: 0.28, 0.97] if all other factors remained constant.

Those pregnant women enrolled on the exited TSFP were 2.47 (AOR: 2.47; 95% CI: 1.35, 4.50) times more likely to have inadequate weight gain related to women who gained adequate weight. If pregnant women increase her parity (number of viable pregnancies) by 1 unit, the odds of inadequate weight gain related to adequate weight gain would be expected to decrease by a factor of 0.18 (AOR: 0.18; 95% CI: 0.05, 0.62) given the other variables in the model were held constant. Those who had no history of alcohol intake during pregnancy were 53% (AOR: 0.47; 95% CI: 0.25, 0.88) less likely to have inadequate weight gain related to adequate weight gain.

### Overadequate weight gain related to adequate weight gain adequacy

For pregnant women in urban relative to rural, the odds of overadequate weight gain to adequate weight gain, it was increased by a factor of 5.22 (AOR: 5.22; 95% CI: 2.43, 11.22), i.e., pregnant women in the urban areas were more likely to hand overadequate weight gain than rural areas as compared with adequate weight gain. The pregnant women enrolled on TSFP were 2.22 (AOR: 2.22; 95% CI: 1.08, 4.57) times more likely to have overadequate weight gain.

## Discussion

Even if GWG is well discourses in developed countries, there is little information in developing countries, which is a neglected public health issue in the era of the double burden of malnutrition with significant short and long-term consequences for both the mother and the fetus. Most of the time, the problem is left unstudied because of difficulties associated with data collection throughout the pregnancy [22]. Besides, working on and addressing this sensitive issue is a straightforward intervention agenda to break the intergenerational effects of malnutrition across life spans.

In this study, most of the pregnant women had adequate weight gain (65.12%); adequate gestational weight gain is essential to avoid adverse pregnancy outcomes. The rate of inadequate and overadequate weight gain was (21.53%) and (13.35%), respectively, as per the IOM recommendation. This study found that adequate weight gain was relatively high, inadequate weight was inline, and overadequate weight gain was lower than the study finding in Canada; the rate of adequate, inadequate, and overadequate weight gain was 49.4%, 17.6%, and 42%, correspondingly [9]. Also, the study finding in Addis Ababa (Ethiopia) [23] and Harari regional state (Ethiopia) [24] shows a different pattern of weight gain rate, which is an inverse in proportion. Moreover, a study in Niger reveals a high prevalence of inadequate gestational weight gain among pregnant women [25], which is similar to these study findings in Ethiopia. The possible justification might be the differences in population characteristics, study settings, dietary habits, time of prepregnancy BMI data collection, lifestyle and access, and follow-up of the health care system.

Inadequate weight gain was related to meal frequency (AOR: 0.52; 95% CI: 0.28, 0.97), enrollment into TSFP (AOR: 2.47; 95% CI: 1.35, 4.50), parity [AOR: 0.18; 95% CI: 0.18 (0.05, 0.62)], and history of taking alcohol (AOR: 0.47; 95% CI: 0.25, 0.88). According to the study conducted in Finnish, there was no difference in parity between the 3 groups of women [6]. Among underweight women, demographic characteristics, such as education less than high school, were positively associated with inadequate weight gain [2,26] owning to TSFP status and meal frequency. Having multiple parties was a potential risk factor for long-term weight gain for 2 reasons. First, primigravida women are at risk of long-term weight gain because they gain the most weight during pregnancy, and high-gestational weight gain by itself is a risk factor for long-term weight gain. Second, women of higher parity (4+) are at risk of long-term weight gain because they gain more weight in association with pregnancy, irrespective of the amount of weight they gain during their pregnancies [27]. Potentially, it might be because of being physically inactive and taking more caloric foods. This situation may not be true for developing countries because of multiple influences, such as reproduction, production, care, and workload. This problem will be more severe when the parity increases.

Gestational weight gain for women who had a history of alcohol consumption was 47% (AOR: 0.47; 95% CI: 0.25, 0.88) more likely to have inadequate weight gain because of the strong influence of behavioral factors, such as smoking and alcohol consumption are also the causes of inadequate weight gain because of economic burden, loss of eating patterns and poor health care [28], which is in line with this study. These behaviors, can lead to the inability to purchase a balanced diet because of economic constraints, which might alter the eating behavior, and health care seeking habits.

Overadequate weight gain during pregnancy was associated with residency (AOR: 5.22; 95% CI: 2.43, 11.22) and TSFP status (AOR: 2.22; 95% CI: 1.08, 4.57). According to the study conducted in Iran, 48.1% of the women in the urban area had weight gains below the IOM recommendation. Also, the underweight rural women gained less weight in the second and the third trimesters of their pregnancy than the urban women [29], which is in line with this study. The possible reason might be increased sedentary behaviors, being physically inactive and consumption of energy-dense and ultraprocessed food in urban areas.

In this study, targeted supplementary feeding increases the odds of overadequate weight gain by 2.22 tomes (AOR: 2.22; 95% CI: 1.08, 4.57). Likewise, the effect of TSFP is associated similarly associated with overadequate weight gain in the study conducted at North Carolina Hospital in Chapel Hill, NC, United States. Women with a history of dieting or restrained eating gained more weight during pregnancy and had higher adequacy of weight gain ratios [30]. More probably, increasing dietary intake and meal frequency raises the probability of consuming more calories than we expend, resulting in a higher likelihood of gaining excess weight. Besides this, it might be because of unhealthy dietary habits, such as eating of junk foods because of erratic behavior of eating during pregnancy.

### Limitation of the study

Primarily, the IOM gestational weight gain recommendation is established for developed countries, which might not be appropriate for developing countries, such as Ethiopia. Furthermore, the prepregnancy weight was measured at the time of conception period, which might affect the prepregnancy BMI because of significant weight gain or loss. Also, weight gain after 9 mo of pregnancy was not taken. Finally, the study is conducted at the community level, so important clinical variables might not be considered, and the recall biases may affect some variables, such as dietary habits.

Furthermore, the extended data collection period prevented us from capturing data on food insecurity, which varies throughout the year because of fluctuations in agricultural productivity. Our data collection began in September [all variables were collected at this time except the outcome variable (weight gain), a time when food insecurity is less likely in rural parts of Ethiopia]. As a result, we could not include food insecurity data in our study.

## Conclusion

In this study, inadequate and overadequate weight gain is significant. The contributing factors to inadequate weight gain were meal frequency per day, TSFP enrollment status, parity, and history of alcohol consumption during pregnancy/near conception time. And on the other side, the contributing factors for overadequate weight gain were residence and TSFP enrollment status.

Health care providers may have to consider parity, residence, and extra meal frequency practice during and before preconception for appropriate weight gain. Also, they must assess and link to the available target supplementary feeding services.

All other stakeholders shall have to emphasize the advocacy of prepregnancy BMI for reproductive age group women for prevention and control of inappropriate weight gains. Also, preconception care is essential to avoid inadequate and overadequate weight gains through lifestyle modification. Overall, nutrition and health education targeting prepregnant women are vital in addition to feeding programs.

## Author contributions

The authors’ responsibilities were as follows – MTE: made the draft of the proposal and acquisition, analysis of data, and the interpretation or discussion results of the manuscript. ADG, BAA, DT, SAT, YA, YYB: worked on the proposal development, analysis, interpretation, discussion, and results, and revised the manuscript; and all authors: read and approved the last version of the manuscript.

## Funding

The study received support from Debre Tabor University during the data collection process.

## Data availability

All the important data are found in the article.

## Conflict of interest

The authors report no conflicts of interest.
